# Plant physiological control of root microbiome assembly and feedback under environmental stress

**DOI:** 10.3389/fmicb.2026.1974097

**Published:** 2026-10-21

**Authors:** Meihong Ge, Qingmian Chen, Yonglian Wang, Yunxiao Han, Faqin Lian, Shuailing Hou, Xiating Chen, Jiajing Liu, Changbo Zhong, Qiaoyan Zhang, Xuefeng Xu

**Affiliations:** 1 Hainan Free Trade Port Biopharmaceutical Industry Chain and Supply Chain Digitalization Research Base, Hainan Vocational University of Science and Technology, Haikou, China; 2 Institute of Environment and Plant Protection, Chinese Academy of Tropical Agricultural Sciences, Haikou, China; 3 Institute of Agricultural Environment and Soil, Hainan Academy of Agricultural Sciences, Haikou, China; 4 Center for Water and Ecology, State Key Laboratory of Regional Environment and Sustainability, School of Environment, Tsinghua University, Beijing, China

**Keywords:** immune gating, microbial feedback, plant stress physiology, plant-microbe interactions, rhizosphere microbiome, root exudation

## Abstract

Root-associated microbiomes contribute to plant nutrition, defense and stress tolerance, but their variable responses across hosts and environments limit the predictability of microbiome-based interventions. This review examines plant physiological regulation of root microbiome assembly and feedback under environmental stress. Evidence from plant and microbial genetics, defined-metabolite assays, synthetic communities and physiological analyses links changes in carbon allocation, nutrient status, hydraulics, redox balance and hormone–immune signaling to root-derived outputs and microbial responses. These outputs influence microbial sensing and resource use, while host immune and metabolic conditions help regulate colonization and persistence. Flavonoid–NodD signaling, strigolactone-induced mycorrhizal fungal responses and coumarin-mediated selection illustrate comparatively well-resolved transitions; the roles of stress-associated purines, nucleotides and compatible solutes remain more context dependent. However, microbial enrichment and metabolite associations alone do not establish plant-controlled recruitment or benefit. Stronger evidence connects plant regulation, microbial response mechanisms and physiological outcomes through causal perturbations. Microbial activity can modify host physiology, although evidence that the altered host state subsequently redirects community assembly remains limited. We integrate these illustrative findings into a framework linking physiological regulation, root outputs, microbial responses, establishment and feedback. This synthesis identifies testable connections for improving microbiome interventions under stress. Translation requires attention to plant carbon and immune costs, competition within resident communities, and validation across host genotypes and environments.

## Graphical abstract

**Figure:**
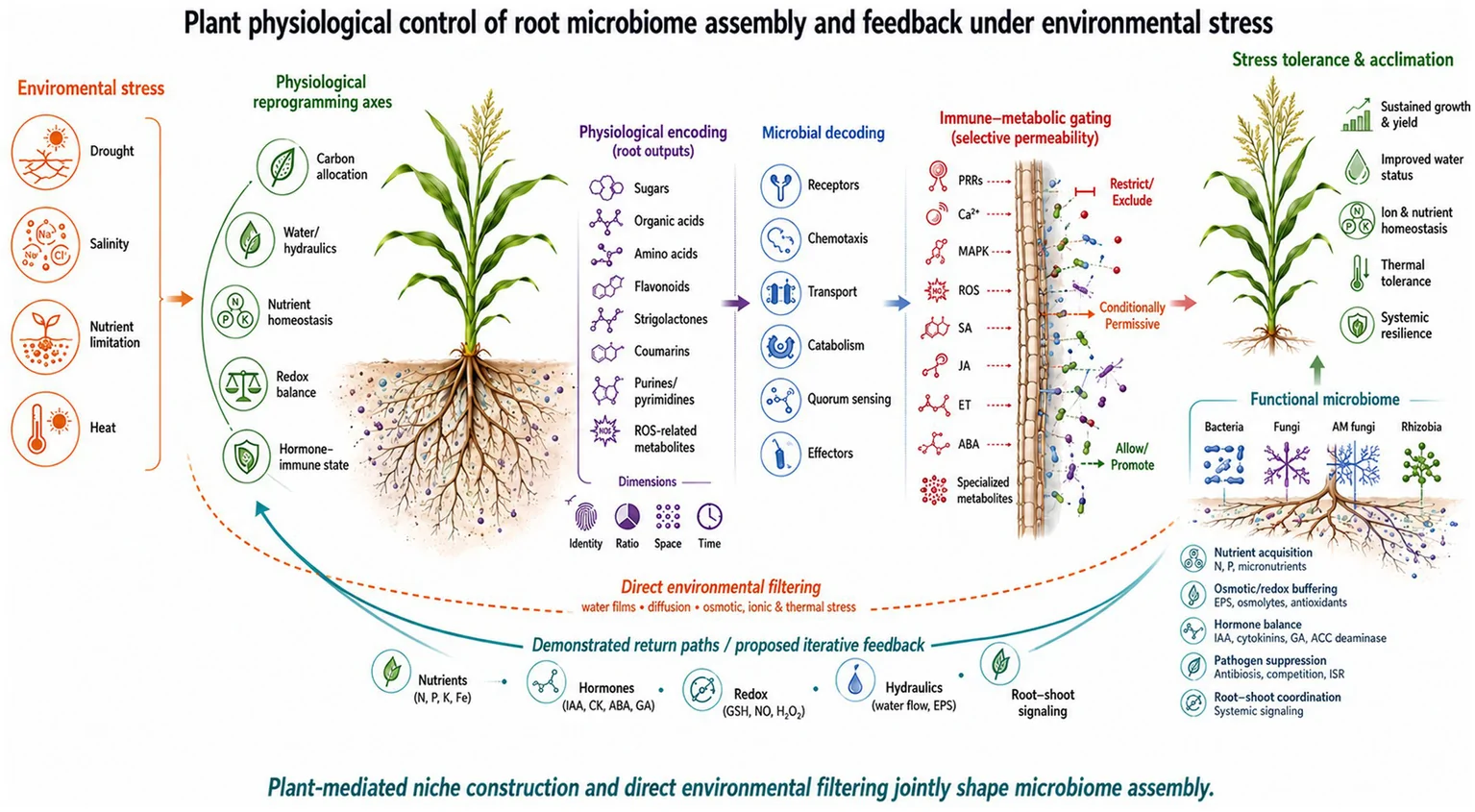

## 1 From microbiome shifts to physiological control

### 1.1 From descriptive microbiome shifts to mechanistic understanding

Plant roots support structured bacterial and fungal communities that contribute to plant nutrition, health and responses to environmental stress. Studies in *Arabidopsis thaliana* established that root-associated communities contain reproducibly enriched lineages and are shaped jointly by soil conditions and host compartment (Lundberg et al., 2012; Bulgarelli et al., 2012). Subsequent work showed that interactions among bacteria, fungi and oomycetes can influence plant survival (Durán et al., 2018). Mechanistic studies have also begun to connect community assembly to plant regulation: phosphate-starvation signaling interacts with immunity through the root microbiota (Castrillo et al., 2017), while changes in exudate chemistry across plant development are associated with microbial substrate preferences (Zhalnina et al., 2018). These advances establish that root microbiome assembly involves both ecological interactions and physiological processes within the host. An unresolved challenge is to explain how these processes connect under environmental stress. A change in microbial abundance may result from direct environmental filtering, altered resource availability, host immune selection or interactions among microorganisms. Likewise, evidence that a metabolite affects microbial growth does not establish that the plant regulates its release under stress, or that the responding microorganisms subsequently improve plant performance. Existing concepts of host filtering, root-exudate-mediated assembly and cry-for-help responses explain complementary parts of this problem, while synthetic communities provide tools for testing causal relationships and feedback studies examine microbial effects on the host (Bulgarelli et al., 2013; Fitzpatrick et al., 2020; Trivedi et al., 2020). However, evidence connecting a defined change in plant state to a microbial response and then to a plant physiological outcome often remains distributed across separate studies or experimental systems. This limits our ability to explain when stress-associated community changes reflect plant-mediated assembly and when they confer a functional benefit.

### 1.2 Root physiological state as an upstream controller of microbiome assembly

Plant physiological state provides a basis for connecting these observations because it links stress perception to changes in the root environment. Environmental stress acts directly on microorganisms and also alters the host conditions that microorganisms encounter. Carbon allocation determines the resources available for rhizodeposition; nutrient status regulates specialized exudation programs; and water status influences root growth, membrane transport and metabolite diffusion. Redox balance and hormone–immune signaling further modify secretion, barrier properties and the conditions permitting microbial establishment. These responses interact, producing root outputs whose composition, amount and spatial and temporal distribution depend on the physiological state of the plant (Bais et al., 2006; Sasse et al., 2018; Vives-Peris et al., 2020; Robert et al., 2025). Following these changes provides a way to examine how plants bias microbial arrival, growth and persistence within the constraints imposed by soil conditions and resident communities. Here, we use this physiological perspective to integrate mechanisms linking environmental stress, root-derived outputs, microbial responses, community establishment and effects on plant performance. We first examine how stress changes root physiology and the production and release of metabolites, then consider how microbial sensing and metabolic traits translate these outputs into responses. We next assess how host immune and metabolic conditions influence establishment and how microbial activity can feed back on plant physiology. Across these transitions, we compare the evidence for individual mechanisms with the evidence connecting them, distinguishing established relationships from proposed extensions and identifying experiments needed to resolve missing links. The resulting framework organizes existing knowledge around the causal connections required to explain plant-mediated microbiome assembly and its physiological consequences (Figure 1).

**Figure 1:**
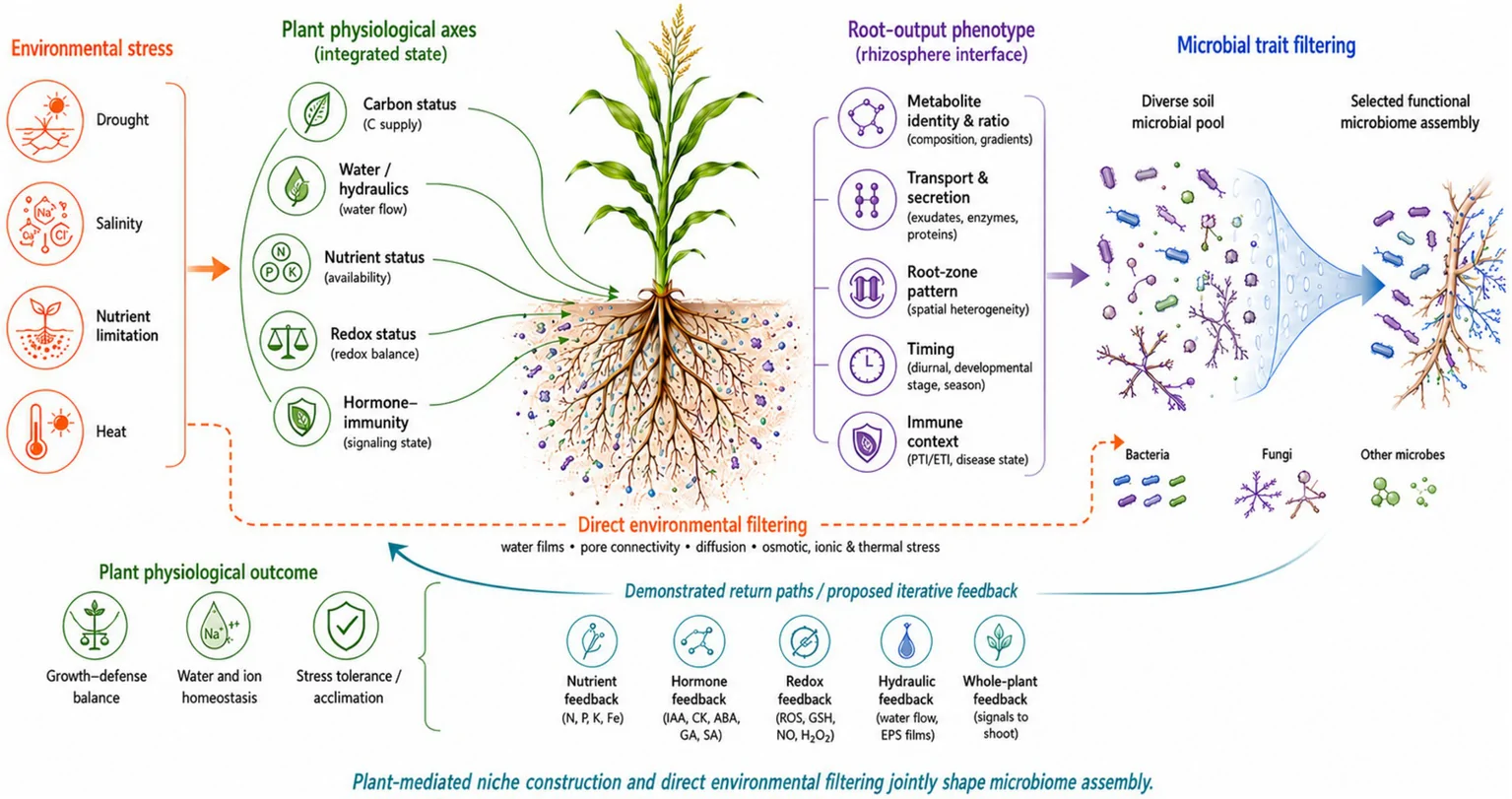
Physiological architecture controlling root microbiome assembly under environmental stress. Environmental stress acts through two concurrent routes: direct environmental filtering of microorganisms and plant-mediated niche construction. Stress reprograms carbon allocation, water and hydraulic status, nutrient homeostasis, redox balance, and hormone–immune state. These axes structure root outputs by identity, mixture, transport, spatial release and timing. Microbial sensing and metabolic traits, followed by immune–metabolic gating, determine colonization, persistence and functional activity. Microbial effects on plant nutrition, hormones, redox state, hydraulics and defense are return paths; an iterative feedback loop is inferred only when the altered plant state is shown to change a subsequent round of assembly.

### 1.3 Scope and literature approach

This review focuses on plant physiological control of root microbiome assembly and feedback under environmental stress. The literature search scope covers publications indexed in Web of Science, PubMed, Google Scholar, and publisher-specific databases as of August 2026. Search combinations included root, rhizosphere, microbiome, drought, salinity, heat, nutrient limitation, pathogen challenge, root exudates, plant hormones, immunity, redox regulation, carbon allocation, microbial sensing, chemotaxis, colonization, community assembly and physiological feedback. Primary studies were prioritized for claims linking plant regulation to microbial responses and plant outcomes. Evidence was organized by the physiological or microbial connection examined, with attention to causal perturbations and measured endpoints. Studies reporting community shifts without mechanistic tests were retained as contextual evidence. The criteria used to distinguish association, plant control, microbial responses and linked physiological effects are described in Section 1.4.

### 1.4 Operational criteria for inferring active microbial recruitment

We distinguish active microbial recruitment from stress-associated enrichment using five causal evidence stages (Table 1). Stage 1 identifies reproducible associations but does not establish plant control. Stages 2 and 3 resolve complementary mechanistic components by demonstrating plant regulation of microbial assembly and microbial decoding of plant-derived outputs, respectively. We reserve a complete active-recruitment claim for Stage 4, in which these components are causally connected through addition, loss-of-function, complementation, strain dropout or rescue to reconstruct the plant-output–microbe–plant-phenotype chain. Stage 5 evaluates whether the resolved mechanism persists in resident communities and across relevant genotypes or environments. These stages describe distinct evidential transitions rather than an automatically cumulative score; therefore, evidence assigned to a body of literature reflects the strongest directly supported transition, and complete-chain claims are made only when the necessary links are experimentally connected.

**Table 1:** Causal evidence stages for demonstrating plant physiological control of functional microbiome recruitment.

Causal evidence stage	Required observation	Preferred experiment	Inference supported	Principal limitation
Stage 1. Reproducible association	Stress-linked change in root output, taxon or microbial gene	Time series; replicated survey; paired multi-omics	Identifies candidates and boundary conditions	Does not separate plant control from leakage or filtering
Stage 2. Plant control	Perturbing plant physiology changes assembly	Host mutant; graft; inducible pathway; exudate transfer	Shows a causal plant contribution	May act indirectly through development or resource allocation
Stage 3. Microbial perception or response	A microbial sensor or metabolic pathway responds	Receptor; chemotaxis; transporter; catabolic mutant	Defines microbial decoding and specificity	Single-strain effects may not predict communities
Stage 4. Chain reconstruction	Manipulation recreates the output-microbe-plant phenotype sequence	Metabolite addition; complementation; dropout; SynCom rescue	Supports active recruitment and function	Simplified systems can inflate establishment and effect size
Stage 5. Ecological validation	The mechanism persists across resident communities and environmental variation	Nonsterile soil; genotypes; sites; seasons; field perturbation	Establishes generality, stability and relevance	Expensive, context dependent and rarely achieved for full chains

## 2 Environmental stress reprograms root physiology

Environmental stress alters root microbiome assembly through coordinated physiological reprogramming rather than through one linear signal. Carbon availability and source-sink relations, water and hydraulic status, nutrient homeostasis, redox balance, and hormone-immune state determine the quantity, composition, timing, and spatial distribution of rhizodeposition. Direct effects of water, ions, temperature, and soil structure on microorganisms occur simultaneously. Mechanistic analysis must therefore partition plant-mediated niche construction from environmental filtering and connect both to measurable plant physiological outcomes (Hart, 2014; Bai et al., 2022; Berg et al., 2016). Environmental stress also acts directly on microorganisms and on the physical path between soil and root. Drought reduces water-film continuity, pore connectivity and solute diffusion; salinity changes osmotic potential and ion activity; and heat alters membrane stability, enzyme activity and survival. These effects can enrich tolerant organisms without a change in plant signaling. Host-dependent enrichment is therefore insufficient evidence of active recruitment. Unplanted-soil controls quantify direct environmental filtering, whereas sterile exudate transfer separates root-output effects from the living root. Split-root and grafting designs distinguish local from systemic plant control, and independently controlled root- and shoot-zone treatments separate direct root-zone stress from shoot-derived physiological signals. Combining these controls with absolute microbial abundance, root-output flux and plant physiological measurements provides the minimum design needed to partition direct filtering, passive access and plant-mediated niche construction.

### 2.1 Carbon allocation and root source-sink relations

Stress changes the carbon economy that sustains both root growth and microbial substrates. Drought and heat reduce photosynthesis, modify phloem delivery, and alter the balance between shoot maintenance, root growth, storage, and rhizodeposition. These changes can shift exudates from growth-associated sugars toward organic acids, amino acids, osmolytes, and specialized metabolites. Exudation is therefore a regulated allocation decision with a physiological cost, not a cost-free invitation to beneficial microorganisms. Collection experiments also show that exudate profiles depend on plant carbon supply, developmental stage, diurnal timing, and the presence of microorganisms (McLaughlin et al., 2023). Under drought, enriched microbial carbohydrate-use and iron-acquisition capacities may reflect the substrate landscape produced by altered source-sink relations, but metabolite-taxon covariance does not establish directed recruitment (Xu et al., 2021; Caddell et al., 2023). Short-term comparisons of planted and unplanted soils further show that plant mediation and direct drought effects can be partly decoupled and crop dependent (Bandopadhyay et al., 2024). Experiments should quantify photosynthesis, root carbon delivery, exudation rate, microbial assimilation, and plant benefit together. The relevant question is how much carbon a plant invests, where and when it is released, which organisms capture it, and whether the returned physiological function exceeds the allocation cost (Jones et al., 2009; Oburger and Jones, 2018; Chaparro et al., 2013; Korenblum et al., 2020). Drought-associated enrichment of monoderm bacteria illustrates why carbon physiology must precede community interpretation. Across sorghum and other grasses, enrichment develops together with changes in plant development and root metabolism, and genome-resolved analyses identify capacities for carbohydrate transport, secondary-metabolite use, and iron acquisition (Xu et al., 2018, 2021). These traits are compatible with drought-modified substrates but do not show that the plant rewards a protective organism. Pipecolic-acid-associated profiles likewise identify a candidate physiological state rather than a private recruitment signal (Caddell et al., 2023). A causal source-sink experiment would combine gas exchange, phloem or isotope tracing, root metabolite flux, exudate transfer, microbial uptake mutants, and plant performance. Such a design can separate reduced carbon supply, regulated redistribution, tissue leakage, and microbial capture of newly fixed carbon.

### 2.2 Water relations, hydraulic status and redox balance

Drought, salinity, and heat converge on water relations, osmotic adjustment, and redox control. Reduced soil water potential lowers root hydraulic conductance, changes aquaporin abundance or activity, constrains cell expansion, and redistributes carbon; salinity adds ion toxicity, whereas heat can uncouple shoot and root-zone temperatures. These states alter root outputs and the physical diffusion environment encountered by microorganisms. Root hair developmental regulators provide host-genetic evidence that drought-responsive root structure can reshape microbiome-mediated protection, including recruitment of Rhizobiaceae with defined stress-alleviating functions (Wang et al., 2024). Symbiotic feedback can also act directly on plant water transport: mycorrhiza-associated changes in root and leaf aquaporin composition accompany faster recovery of stomatal conductance and CO₂ assimilation after drought, and LjNIP1;5 links AM colonization with drought tolerance in *Lotus japonicus* (Paluch-Lubawa and Polcyn, 2024; Zou et al., 2024). ROS connects hydraulic and osmotic stress to immunity and microbial filtering. Localized ROS is informative, whereas sustained excess damages both host and microbiota. A water-deficit recruitment claim is strongest when root-zone temperature, hydraulic conductance, aquaporins, osmotic potential, ROS status, exudation, colonization, and plant performance are measured in the same causal design (Xu and Coleman-Derr, 2019; Naylor et al., 2017; Santos-Medellín et al., 2017; Santos-Medellín et al., 2021). Plant-mediated and direct environmental effects should be separated experimentally. Unplanted soil controls reveal microbial responses to low moisture or high temperature, whereas split-root, grafting, and independently controlled root- and shoot-temperature treatments isolate systemic plant contributions. Root hydraulic conductance and aquaporin activity determine water delivery, but they also affect root growth, cell-wall properties, and solute transport. Osmotic adjustment changes compatible-solute pools that may protect both plant cells and microbial responders. Meanwhile, soil drying reduces pore connectivity and metabolite diffusion, potentially preventing a chemically responsive strain from reaching the root. A mechanistic model must therefore integrate root water uptake, soil physics, root anatomy, redox status, and microbial movement. This integration prevents drought-associated enrichment from being interpreted as active recruitment when it can be explained by desiccation tolerance or altered dispersal (Affortit et al., 2023; Gupta et al., 2020).

### 2.3 Nutrient homeostasis as a microbiome-control axis

Nutrient limitation provides the clearest examples of physiological demand being encoded into microbial assembly. Under phosphate limitation, the phosphate-starvation response remodels root architecture and immunity and induces strigolactone release. Strigolactones activate AM fungal metabolism and hyphal branching, increasing the probability of contact and phosphorus exchange (Akiyama et al., 2005; Besserer et al., 2006). Systemic phosphate status subsequently restricts colonization when the carbon cost exceeds nutrient demand (Balzergue et al., 2011). In legumes, nitrogen status and flavonoid-NodD signaling regulate rhizobial entry and reshape the wider exudate and bacterial environment (Zgadzaj et al., 2016; Tao et al., 2024). Iron deficiency activates MYB72-dependent coumarin secretion, which mobilizes iron and selects bacteria through tolerance and metabolism (Stringlis et al., 2018; Voges et al., 2019; Harbort et al., 2020). These systems expose a central physiological trade-off: AM fungi and rhizobia require photosynthate, and exudation itself consumes carbon. Recruitment is therefore an optimization problem linking nutrient return, carbon expenditure, growth, and defense, not a process in which greater colonization is automatically better (Oldroyd, 2013; MacLean et al., 2017; Rajniak et al., 2018). The three nutrient systems also differ in architecture and physiological return. AM fungi explore soil through an external hyphal network but can consume a substantial fraction of plant-assimilated carbon. Rhizobia exchange fixed nitrogen within a specialized organ whose initiation and maintenance are tightly controlled by the host. Coumarin-responsive bacteria modify iron chemistry without forming a dedicated symbiotic structure. The phosphate-starvation response also changes bacterial assembly and immunity independently of AM symbiosis, demonstrating that nutrient homeostasis can operate through several microbial routes (Castrillo et al., 2017; Finkel et al., 2019). Comparisons should quantify nutrient uptake per unit carbon invested and test whether colonization is downregulated when demand is satisfied. These measurements would convert the idea of recruitment cost into an explicit physiological balance among nutrient gain, photosynthate expenditure, root growth, and defense.

### 2.4 Hormone-immune integration

The ABA, SA, JA, and ET are first plant physiological regulatory networks and only secondarily microbiome regulators. SA pathway perturbations provide direct evidence for taxon-selective root filtering, and jasmonate effects vary among plant compartments (Lebeis et al., 2015; Carvalhais et al., 2013; Liu et al., 2017). Ethylene restricts early Nod-factor signaling, whereas auxin contributes to rhizobial infection and root development (Oldroyd et al., 2001; Breakspear et al., 2014). IBM1 dysfunction links epigenetic autoimmunity with altered community assembly, demonstrating that maximal defense is not optimal for microbiome homeostasis (Lv et al., 2022). ABA is central to stomatal, hydraulic, root-growth, and carbon responses under drought, but direct receptor-level evidence that ABA acts as a microbial recruitment cue remains limited. It should therefore be treated as an upstream regulator of exudation and immune context. Hormone-immune integration defines the physiological permissiveness within which microbial decoding occurs. Hormonal interpretation also requires spatial and temporal resolution. SA or JA perturbation can change specific taxa without uniformly reducing microbial load, and the effect may differ between rhizosphere, root surface, endosphere, and shoot. Constitutive immune activation can be as disruptive as immune deficiency: IBM1 dysfunction produces autoimmunity and reorganizes the Arabidopsis root microbiome (Lv et al., 2022). ABA illustrates a different limitation. Its established roles in stomatal closure, root hydraulic adjustment, and carbon allocation make it a plausible upstream controller, but these pleiotropic effects can change the recruitment environment without ABA being sensed by microbes. ABA pathway mutants should therefore be paired with sterile exudate transfer, microbial response assays, spatial colonization, and physiological rescue before a direct recruitment function is claimed.

## 3 Physiological encoding of root microbial niches

Physiological encoding is the conversion of plant state into root outputs that microorganisms can encounter and interpret. Compound identity is only one component. Biosynthesis, transporter activity, secretion site, concentration, mixture, timing, developmental stage, and immune context jointly determine the niche. Root-derived compounds may function as directional cues, regulatory ligands, selective resources, redox modifiers, or antimicrobials; the appropriate term depends on demonstrated mechanism. Figure 2 and Table 2 organize these components from plant regulation to microbial decoding and plant physiological outcome.

**Figure 2:**
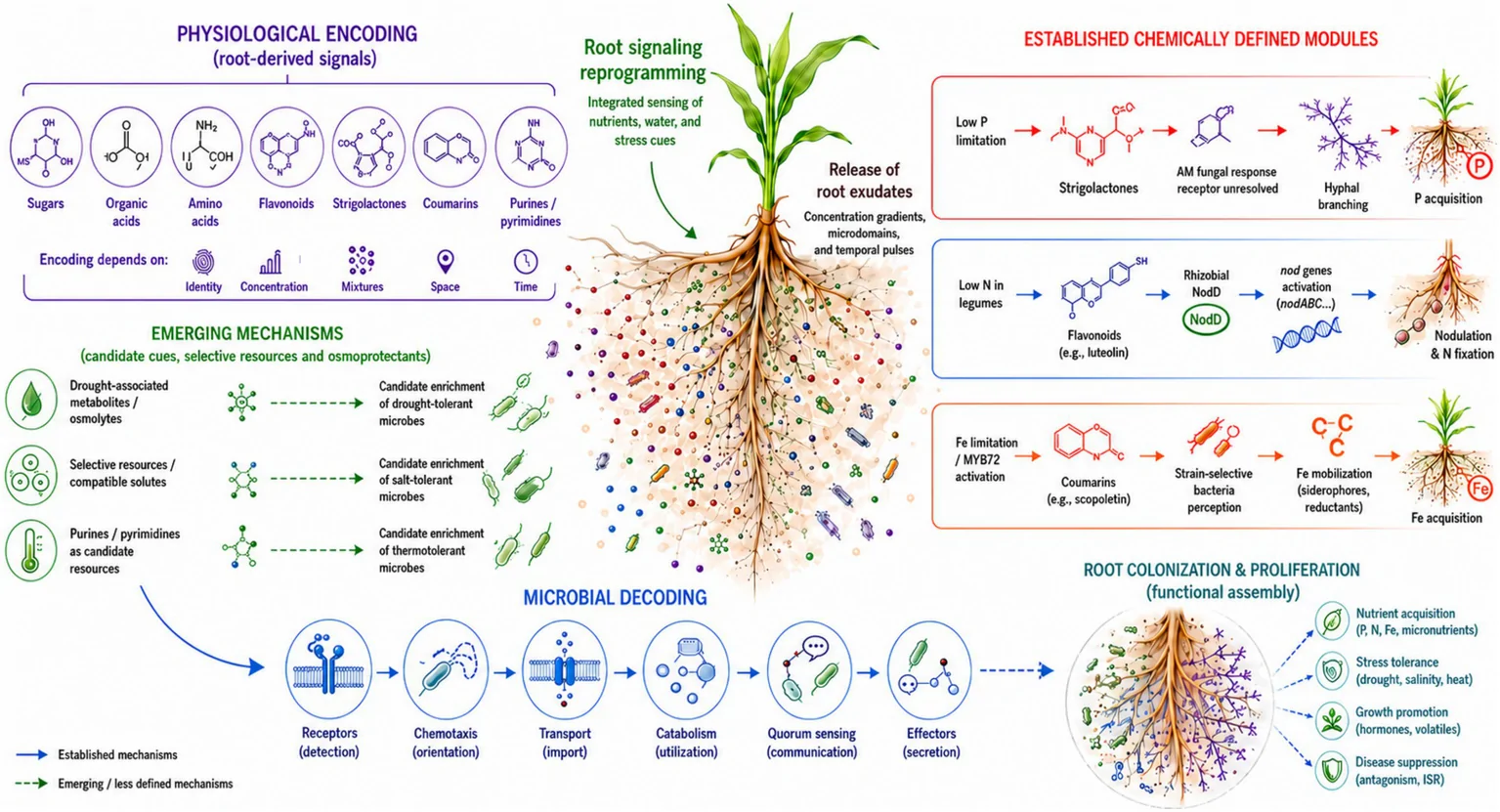
Physiological encoding and microbial decoding of root-derived outputs. Plant physiological state regulates the biosynthesis, transport and spatial or temporal release of root outputs. Flavonoid–NodD signaling, strigolactone-induced arbuscular mycorrhizal (AM) fungal responses and coumarin-mediated selection are comparatively well-resolved modules. The AM fungal receptor for strigolactones remains unresolved; D14/KAI2 are plant signaling components and are not assigned as fungal receptors. Stress-associated purines, pyrimidines and compatible solutes are shown as emerging candidate cues, selective resources or osmoprotectants. Solid arrows denote established transitions; dashed arrows denote emerging or incompletely resolved transitions.

**Table 2:** Representative physiological signaling modules linking plant stress status to microbiome recruitment.

Stress/physiological state	Plant regulatory pathway	Root output	Microbial decoder	Colonization response	Plant physiological outcome	Strongest causal evidence stage(s)
Low N in legumes^a^	N status; symbiosis signaling	Transported flavonoids	NodD; nod genes	Rhizobial entry and nodulation	N acquisition for photosynthate	Stages 4–5
Low P^b^	PSR; strigolactone biosynthesis	Strigolactones	Experimentally linked mitochondrial activation and hyphal branching; receptor identity unresolved	Hyphal branching and root encounter	P gain versus carbon cost	Stages 4–5*
Fe limitation^c^	Fe-deficiency / MYB72	Coumarins	Tolerance, transport and metabolism	Strain-selective assembly	Fe mobilization and nutrition	Stage 4
Drought^d^	ABA; carbon, hydraulic and redox state	Reprogrammed metabolites; osmolytes	Transport, catabolism and stress tolerance	Conditional enrichment and persistence	Hydraulic, osmotic and redox support	Stages 1–3
Salinity^e^	Osmotic, ionic and redox regulation	Purines; compatible solutes	Selective use; antioxidant accommodation	*Pseudomonas* enrichment	Ion/redox balance and growth	Stage 4
Heat^f^	Thermal and systemic carbon state	Purines and pyrimidines	Metabolic use by bacterial/fungal responders	Protective colonization	Thermotolerance and disease suppression	Stages 3–4
Root development^g^	SWEET distribution; root-zone physiology	Spatial sugars and metabolites	Chemotaxis, uptake and catabolism	Longitudinal organization	Local growth and nutrient exchange	Stages 3–4
Immune activation^h^	SA-JA-ET-ABA calibration	Immune and exudate context	Recognition, effectors and tolerance	Restriction or accommodation	Growth-defense balance	Stages 2–4
Pathogen attack^i^	Systemic defense and coumarin pathways	Defense-regulated exudates	Consortium response and cooperation	Disease-suppressive assembly	Primed defense and pathogen suppression	Stages 3–4
Multiple stresses^j^	Integrated carbon, hormone and redox state	ROS, phenolics and resource shifts	Detoxification, biofilms and competition	Context-dependent persistence	Conditional stress buffering	Stages 1–3

AM, arbuscular mycorrhizal; ABA, abscisic acid; ET, ethylene; JA, jasmonic acid; SA, salicylic acid; SynCom, synthetic community.

Causal evidence stages indicate the strongest directly supported transition represented by the cited studies and should not be interpreted as cumulative. Ranges indicate heterogeneity in the available evidence and do not imply that all preceding transitions were demonstrated within a single experimental system. Stage 1, reproducible association; Stage 2, plant control; Stage 3, microbial decoding or experimentally linked microbial response; Stage 4, causal chain reconstruction; Stage 5, ecological validation. For low-P responses, mitochondrial activation and hyphal branching provide Stage 3 evidence despite the absence of an identified receptor; Stages 4–5 therefore refer to later experimentally supported transitions and should not be interpreted as evidence of receptor identification. *Low P row indicates that this designation does not imply identification of the fungal strigolactone receptor. Mitochondrial activation and hyphal branching provide Stage 3 evidence despite the absence of an identified receptor, whereas Stages 4–5 refer to later experimentally supported transitions in the cited studies. The causal evidence stages are not cumulative.

Representative supporting studies: ^a^Sugiyama et al. (2007), Oldroyd (2013), and Tao et al. (2024); ^b^Akiyama et al. (2005), Besserer et al. (2006), Balzergue et al. (2011), and Genre et al. (2013); ^c^Stringlis et al. (2018), Voges et al. (2019), and Harbort et al. (2020); ^^d^^Naylor et al. (2017) and Xu et al. (2018, 2021); ^e^Zheng et al. (2024); ^f^Liu et al. (2025); ^g^Loo et al. (2024); ʰLebeis et al. (2015), Liu et al. (2017), and Lv et al. (2022); ^i^Berendsen et al. (2018), Vismans et al. (2022), and Yang et al. (2023); ʲrepresentative evidence is discussed in Sections 2–6 because the effects of multiple stresses are strongly context dependent.

### 3.1 Biosynthesis, transport and spatial release of root signals

A released metabolite should not be treated as an unregulated pool. Signal production and export are physiological control points. An ATP-binding cassette transporter involved in soybean genistein secretion links plant transport directly to rhizobial NodD activation (Sugiyama et al., 2007). Along the Arabidopsis root axis, SWEET sugar transporters generate spatial differences in microbial colonization, showing that release location can be as important as compound identity (Loo et al., 2024). Developmental and diurnal variation further changes exudate abundance over hours and across root zones (McLaughlin et al., 2023). Stress may change total flux, ratios among compounds, secretion from specific cell types, or the duration of exposure. These alternatives require different experiments. Biosynthetic mutants test production, transporter mutants test export, microfluidic and imaging approaches test location and timing, and isotope tracing distinguishes plant allocation from microbial transformation. Transport-controlled signaling provides a mechanistic bridge from whole-plant carbon or nutrient status to the microscale chemical fields that organize colonization (Hemelda and Noutoshi, 2025). Measurement itself can obscure encoding. Long hydroponic collection periods integrate several circadian phases, allow microbial consumption, and can accumulate compounds that would never reach the same concentration in soil. Sterile collection removes microbial transformation but also changes plant growth and exudation. Soil minerals adsorb metabolites and create steep microscale gradients. Short collection windows, *in situ* probes, and isotope tracing are therefore needed to distinguish secretion rate from standing concentration. Root anatomy provides another source of heterogeneity: tips, elongation zones, mature tissues, root hairs, and lateral-root junctions differ in permeability and transporter expression. A biologically relevant output should be defined by flux from a specific tissue into a specified physical environment, not by the concentration recovered from a pooled root system.

### 3.2 Chemically defined signaling systems

Three systems provide the strongest templates for causal analysis. In legumes, nitrogen demand promotes regulated flavonoid release; particular flavonoids bind rhizobial NodD regulators, activate nod genes, and initiate Nod-factor-dependent root-hair and nodule development. In mycorrhizal hosts, phosphate starvation induces strigolactones that activate AM fungal mitochondria and hyphal branching before contact; fungal chitin oligomers then engage host calcium signaling, and systemic phosphate and carbon status regulate subsequent colonization (Akiyama et al., 2005; Besserer et al., 2006; Genre et al., 2013). Under iron deficiency, MYB72-dependent coumarins mobilize Fe and select bacteria through strain-specific tolerance, transcription, and metabolism (Stringlis et al., 2018; Voges et al., 2019; Harbort et al., 2020). Each case can be evaluated using the same sequence: physiological trigger, biosynthesis, transport or release, microbial decoder, colonization response, plant physiological consequence, and causal evidence stage. This template separates a defined signaling mechanism from a compound merely associated with microbial enrichment (Harrison, 2005).

### 3.3 Stress-induced primary metabolites as emerging signals

Purines, pyrimidines, pipecolic-acid-associated profiles, compatible solutes, and related primary metabolites are emerging components of stress-dependent recruitment. Salt-stressed wild soybean accumulated purines in roots and exudates, and genetic, multi-omics, and inoculation experiments connected purine-responsive *Pseudomonas* with plant growth under salinity (Zheng et al., 2024). Heat-stressed *Panax notoginseng* enriched purines and pyrimidines that recruited bacterial and fungal responders, with partial transfer across hosts (Liu et al., 2025). Water-stressed wheat exudates contained more choline and glycine betaine; these compounds induced quaternary-ammonium transport and catabolism in *Pseudomonas synxantha* and supported rhizosphere competence under osmotic stress (Bhattacharyya et al., 2025). These examples show how a metabolite can function as a selective resource and microbial stress protectant without necessarily being a dedicated receptor ligand. They should be labeled emerging until plant control, microbial decoding, chain reconstruction, and ecological generality are demonstrated.

### 3.4 Signal identity is not enough: concentration, mixtures, space and time

Signal identity does not equal recruitment specificity. A compound may simultaneously act as an attractant, nutrient, antimicrobial, or redox-active molecule. Its effect depends on concentration, ratio to other exudates, spatial release, stress duration, developmental and circadian state, microbial receptors and transporters, catabolic capacity, community interactions, and host immunity. A layered model predicts that combinations and temporal trajectories can be more informative than single compounds. Spatially organized sugar transport provides one mechanistic entry point (Loo et al., 2024), while diurnal exudation dynamics show that bulk collection can average biologically distinct windows (McLaughlin et al., 2023). Experiments should combine exudate metabolomics with flux measurements, imaging, microsensors, and spatial transcriptomics. Because pathogens and mutualists may share chemotactic or metabolic capacities, manipulating a single metabolite can alter the accessibility of multiple microbial niches simultaneously. Recruitment specificity is therefore not an intrinsic property of a single compound, but an emergent outcome of its concentration and chemical context, spatial and temporal release patterns, microbial sensing and metabolic traits, and the host immune-metabolic state. This multidimensional view has important implications for experimental design. Dose–response curves should include mutualists, commensals, pathogens, and non-responders, because a compound can widen several niches simultaneously. Mixture experiments can test whether one metabolite modifies perception or use of another. Spatial reporters can determine whether a response occurs before contact, at the root surface, or after entry. Temporal perturbations can separate an early directional cue from a later growth substrate. Finally, the same chemical mixture should be tested under contrasting immune states and resident communities. These factorial designs are more demanding than one-compound assays, but they reveal whether specificity resides in the molecule, the plant physiological context, the microbial decoder, or their interaction.

## 4 Microbial decoding of plant physiological signals

Root outputs influence assembly only when microorganisms detect, access, or exploit them. Microbial decoding includes direct receptor-mediated signaling, chemotaxis, transport-coupled sensing, catabolic induction, population-level communication, and effector-mediated accommodation. These mechanisms convert a plant-derived molecule into movement, transcription, metabolism, cooperation, or altered host response. Their specificity varies from dedicated NodD-flavonoid recognition to broad growth advantages on sugars or organic acids (Venturi and Keel, 2016).

### 4.1 Receptor-mediated sensing and chemotaxis

Chemotaxis is often the first microbial decision point. Root exudate components are detected by methyl-accepting chemotaxis proteins and relayed through CheA-CheY signaling to alter flagellar behavior. In *Bacillus amyloliquefaciens* SQR9, defined cucumber exudate compounds were linked to specific chemoreceptors and colonization (Feng et al., 2018). Organic acids also alter chemotaxis, biofilm formation, and root association (Zhang et al., 2014). Chemotaxis is not intrinsically beneficial: *Ralstonia solanacearum* requires it for competitive colonization and virulence (Yao and Allen, 2006). Dedicated transcriptional receptors provide stronger molecular specificity. Rhizobial NodD proteins bind flavonoids and activate nod genes, whereas subsequent Nod-factor signaling changes both host development and the broader root niche. Thus, attraction must be followed by metabolic compatibility, competition, and immune-metabolic gating before stable colonization can be inferred (Rudrappa et al., 2008; Liu et al., 2024).

### 4.2 Transport, catabolism and metabolic decoding

Transporters and catabolic pathways allow microorganisms to decode metabolites even when no classical receptor is known. Bacteria that import, detoxify, or degrade a root compound gain a conditional advantage and may simultaneously change the compound experienced by neighboring strains. Tomato-associated *Sphingobium* catabolizes toxic steroidal glycoalkaloids, converting a defensive metabolite into a colonization niche (Nakayasu et al., 2023). Coumarin-responsive *Pseudomonas* activates stress, transport, and metabolic programs (Voges et al., 2019; Harbort et al., 2020), and water-stress exudates induce quaternary-ammonium uptake and catabolism in *Pseudomonas* (Bhattacharyya et al., 2025). Microbial enrichment may therefore reflect receptor signaling, chemotaxis, osmoprotection, carbon or nitrogen use, detoxification, or cross-feeding. Mutants in transport and catabolism are essential for identifying which decoding mode operates in a community.

### 4.3 Quorum sensing and community-level communication

After arrival, microorganisms coordinate attachment, biofilm formation, secondary metabolism, and competition. Quorum-sensing molecules can alter plant gene expression, while plant metabolites can mimic, inhibit, or redirect microbial communication (Venturi and Keel, 2016). Soil adsorption, degradation, and quorum quenching make culture concentrations poor proxies for intact rhizospheres, so community-level claims require spatial reporters and signal or receptor mutants. Volatile cues extend communication beyond the immediate root and can influence neighboring plants or systemic resistance (Kong et al., 2021; Naznin et al., 2014). Quorum sensing is therefore one decoding layer within resource gradients and interaction networks, not an independent explanation for recruitment.

### 4.4 Effectors and colonization accommodation

Microbial effectors can redirect host signaling and determine accommodation. Rhizobial type III secretion systems can modify or partly bypass canonical Nod-factor requirements (Okazaki et al., 2013). Beneficial and commensal colonizers also manage immune recognition, often through transient and spatially restricted accommodation rather than permanent suppression. *Trichoderma* colonization alters early defense and antioxidant programs before a stable interaction develops (Brotman et al., 2013), while *Colletotrichum tofieldiae* benefits the host specifically under phosphate deficiency (Hiruma et al., 2016). Effector function depends on host genotype, microbial background, tissue, and physiological context; the same machinery may promote infection, restrict host range, or stabilize mutualism. Multispecies tests are needed to separate improved host entry from altered competition or community-mediated immunity.

## 5 Immune-metabolic gating of root microbiome assembly

Root microbiome assembly requires calibrated immunity rather than a binary barrier. Pattern-recognition receptors, calcium and MAPK signaling, ROS, hormone networks, and specialized metabolites influence where microorganisms enter, whether they persist, and how their activity affects the host. Because nutrition, redox chemistry, and defense share metabolites such as coumarins and ROS, this selection system is best described as immune-metabolic gating. The gate changes colonization probabilities; it does not automatically admit mutualists or exclude all pathogens (Teixeira et al., 2019; Cui et al., 2015). The interacting recognition, hormonal, and metabolic filters that define this gate are integrated in Figure 3.

**Figure 3:**
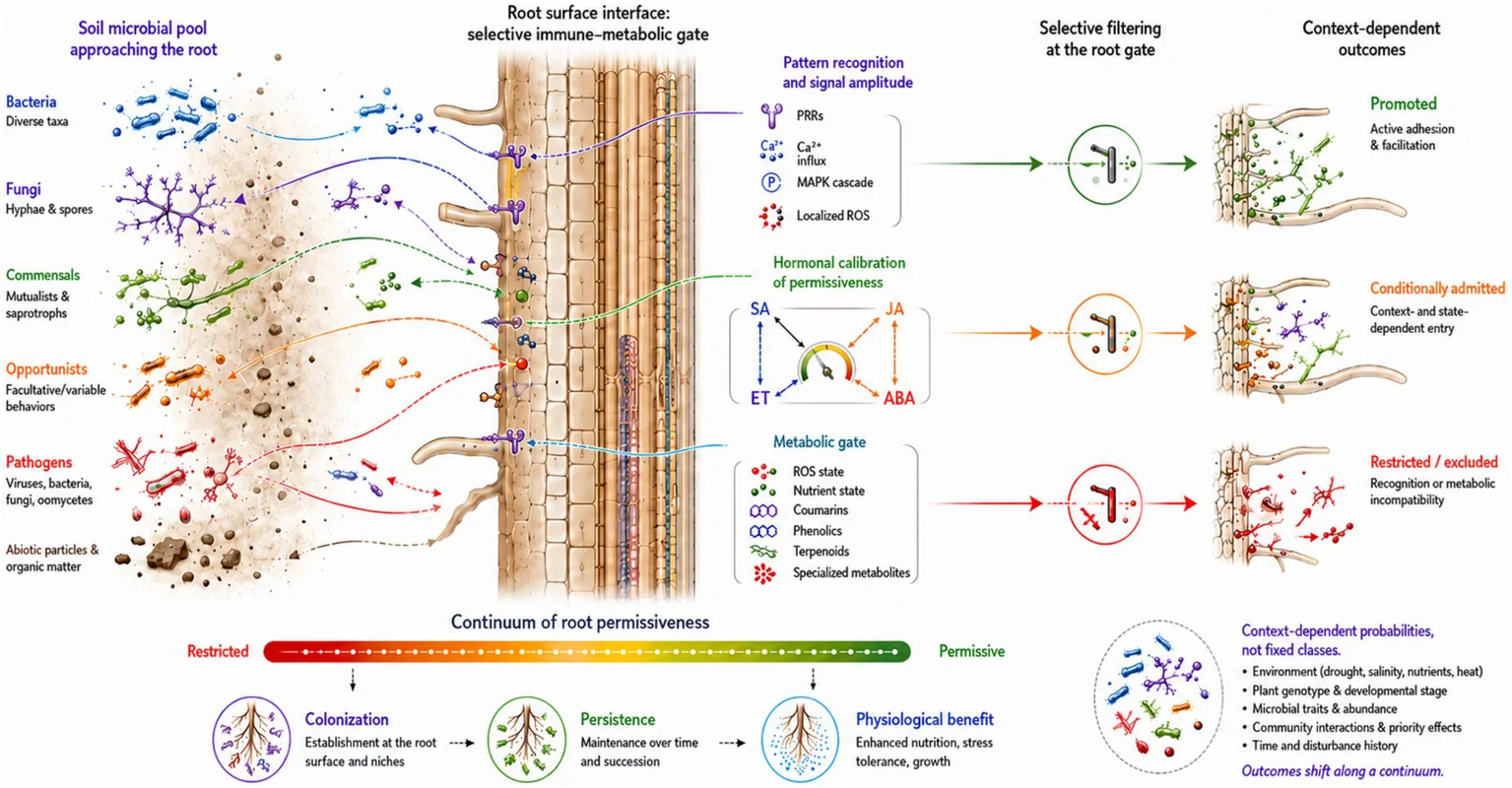
Immune–metabolic gating of root microbiome assembly. Pattern recognition and signal amplitude establish an initial gate; hormonal calibration and metabolic state then modify microbial permissiveness. These layers produce context-dependent probabilities of colonization, persistence and physiological benefit rather than fixed microbial classes. PRR, pattern-recognition receptor; MAPK, mitogen-activated protein kinase; ROS, reactive oxygen species; SA, salicylic acid; JA, jasmonic acid; ET, ethylene; ABA, abscisic acid; Ca^2+^, calcium ion. No asterisk or significance symbol is used in the figure.

### 5.1 Pattern recognition establishes the first gate

Pattern-recognition receptors detect conserved microbial features and activate calcium, MAP kinase, ROS, and transcriptional responses. Because these features are shared by pathogens and commensals, recognition alone cannot determine outcome. Spatial restriction and signal amplitude are critical. Root zones differ in cell differentiation, barrier properties, nutrient leakage, and immune competence. Sugar transporter activity can spatially organize microbial colonization along the root axis, linking developmental anatomy with resource availability and immune exposure (Loo et al., 2024). Microbial communities also modulate immune output. Cross-kingdom interactions among bacteria, fungi, and oomycetes contribute to *Arabidopsis* survival and can suppress the detrimental activity of individual community members (Durán et al., 2018). The host consequently filters not only individual taxa but also interaction networks. A microorganism that appears neutral in isolation may become beneficial by restraining a pathogen or harmful by disrupting protective competition.

### 5.2 Hormonal calibration determines permissiveness

SA-dependent immunity is a causal determinant of root bacterial colonization. SA pathway perturbations alter the abundance of specific taxa, but do not cause uniform community exclusion (Lebeis et al., 2015). Jasmonate activation likewise changes rhizosphere composition, and its effects vary across plant compartments (Carvalhais et al., 2013; Liu et al., 2017). These results support a multidimensional hormone gate. SA, JA, and ethylene reshape microbial access through changes in defense gene expression, root development, nutrient allocation, and exudation. Constitutive immune activation can be as disruptive as immune deficiency. IBM1 dysfunction causes autoimmunity and reorganizes the *Arabidopsis* root microbiome (Lv et al., 2022). This phenotype illustrates that microbiome homeostasis depends on calibrated defense rather than maximal defense. It also provides a mechanistic link between chromatin regulation and community assembly. Epigenetic states can change the baseline immune environment that microorganisms experience, potentially generating persistent microbiome differences without altering exudate biosynthetic genes directly.

### 5.3 ROS and specialized metabolites form a metabolic gate

The ROS production is a rapid defense response, but it also shapes symbiotic entry, cell-wall remodeling, and microbial competition. Excessive ROS damages both host and microbiota, whereas localized ROS can restrict microbial spread or signal developmental transitions. Beneficial colonizers often combine ROS tolerance with the capacity to modulate plant antioxidant systems. The *Trichoderma*-plant interaction under salinity illustrates this reciprocal regulation (Brotman et al., 2013). Specialized metabolites add taxonomic and functional selectivity. Coumarins can suppress sensitive strains while supporting organisms that tolerate or metabolize them (Voges et al., 2019; Harbort et al., 2020). These compounds operate at the boundary between nutrition and immunity. By changing iron availability, redox chemistry, and microbial growth simultaneously, they refine the community that passes through the initial immune gate. Similar principles likely apply to benzoxazinoids, terpenoids, and phenolics, although causal reconstruction is less complete for many of these metabolites.

### 5.4 Systemic stress signaling and the “cry-for-help” response

Aboveground attack can induce belowground recruitment of protective microorganisms. Foliar pathogen challenge caused Arabidopsis to assemble a disease-suppressive bacterial consortium, and reconstruction showed protection in a subsequent plant generation (Berendsen et al., 2018). Tomato RIN-dependent exudation and pathogen-induced coumarin legacies provide additional links from systemic regulation to rhizosphere function (Yang et al., 2023; Vismans et al., 2022). These examples define the “cry-for-help” response as systemically induced physiological gating: stress perception changes whole-plant signaling and root outputs, microbes decode the altered niche, and microbial functions feedback on defense. The response is not automatically beneficial; growth costs, pathogen manipulation, and exclusion of useful commensals remain possible. Necessity, sufficiency, host benefit, and persistence across soils are required to distinguish functional recruitment from a coincident community shift (Mendes et al., 2011; Pieterse et al., 2014; Kwak et al., 2018).

## 6 Microbiome feedback on plant physiology and stress tolerance

Microbiome assembly is physiologically meaningful only when microbial activity feeds back on the plant. The relevant endpoints are not lists of enriched taxa but changes in nutrient and ion homeostasis, hormone balance, redox status, osmotic adjustment, hydraulics, growth-defense allocation, and root-shoot coordination. The same microbial lineage may be neutral under favorable conditions and beneficial or costly under stress, so feedback must be measured in the physiological context that generated recruitment (Berendsen et al., 2012; Lugtenberg and Kamilova, 2009). The convergence of stress-specific root outputs on shared microbial functions and plant physiological feedback is compared in Figure 4.

**Figure 4:**
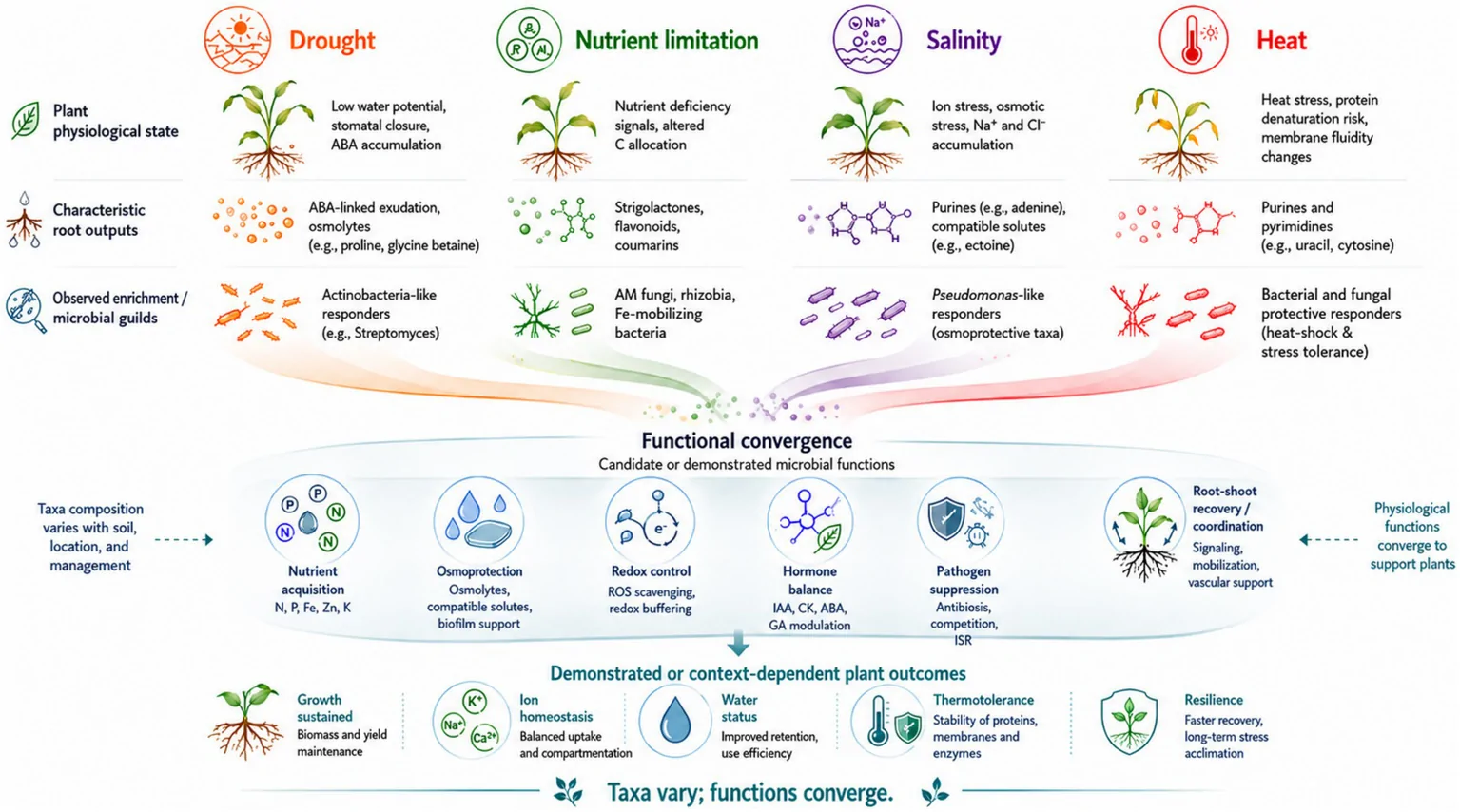
Stress-associated microbiome patterns and context-dependent plant outcomes. Drought, nutrient limitation, salinity and heat are compared across plant physiological state, root outputs, observed microbial enrichment, candidate or demonstrated microbial functions and plant outcomes. Taxonomic enrichment does not by itself establish active recruitment, functional activity or plant benefit. Solid arrows indicate better-supported transitions; dashed or qualified labels indicate associative or context-dependent evidence. Outcomes are described as tolerance, acclimation, resilience or recovery according to the experimental timescale. PSR, phosphate-starvation response; MYB72, MYB domain protein 72; ISR, induced systemic resistance; IAA, indole-3-acetic acid; ACC, 1-aminocyclopropane-1-carboxylic acid; GA, gibberellic acid; GSH, glutathione; EPS, extracellular polymeric substances.

### 6.1 Nutrient acquisition and ion homeostasis

Microbial nitrogen fixation, phosphorus acquisition, iron mobilization, siderophore production, and nutrient transformation affect plant physiology only when they change nutrient uptake, allocation, or deficiency responses. Rhizobia exchange fixed nitrogen for photosynthate, AM fungi extend the soil exploration volume in return for carbon, and coumarin-responsive microbiota improve iron nutrition under limiting conditions (Harbort et al., 2020). These benefits are conditional on host demand and carbon cost. The plant can suppress AM colonization when phosphorus is sufficient, and a siderophore may either improve host Fe acquisition or intensify competition. Physiological feedback should therefore be evaluated through nutrient flux, ion content, transporter activity, growth, and carbon expenditure rather than through microbial functional genes alone (Hacquard et al., 2015). Functional feedback can be reciprocal. Microbial nutrient acquisition changes plant demand signals, and the plant then adjusts exudation, transporter activity, and symbiotic sanctioning. Improved phosphate or iron status can suppress the upstream starvation program that initially favored microbial association. Conversely, a carbon-expensive symbiosis may reduce growth when nutrient return is small. Ion homeostasis under salinity adds another layer because microbial effects on Na^+^, K^+^, and micronutrient balance can change osmotic potential and hormone signaling. Experiments should follow this feedback over time rather than compare only inoculated and uninoculated endpoints. Repeated measurements of nutrient status, root outputs, colonization, and carbon allocation can show whether microbial activity closes a stabilizing loop, triggers negative feedback, or creates a persistent cost.

### 6.2 Hormonal homeostasis and growth-defense balance

Microbial ACC deaminase, auxin, volatiles, and immune-active metabolites can shift hormone homeostasis and growth-defense allocation. ACC deaminase lowers the ethylene precursor 1-aminocyclopropane-1-carboxylate, and gene-level evidence links this activity to plant growth promotion (Herpell et al., 2023). Microbial auxin can alter lateral-root formation, whereas SA- and JA-active communities can prime defense at a cost to growth. The relevant endpoint is not the presence of a microbial hormone-related gene but a host response measured through hormone pools, signaling reporters, root and shoot growth, and defense performance. Because hormone effects depend on dose, tissue, and developmental stage, a microbial output that promotes growth in one context may restrict symbiosis or increase susceptibility in another (Glick, 2014). Hormonal feedback is especially sensitive to context. Reduced ethylene can sustain root elongation during stress but can also alter nodulation, immunity, and senescence. Microbial auxin may enlarge the absorptive root system at low concentrations yet inhibit primary-root growth at higher concentrations. Volatile or immune-active metabolites can prime systemic defense, but constitutive priming can divert carbon from growth. A convincing mechanism therefore requires a microbial gene or metabolite perturbation, a corresponding change in host hormone signaling, and rescue of the predicted plant phenotype. Measuring only microbial hormone production in culture is insufficient. Host reporters, hormone quantification, tissue-specific growth, and pathogen or stress challenges are needed to place microbial activity within the plant’s own regulatory network.

### 6.3 Redox regulation, osmotic adjustment and stress protection

Stress-protective microbiota can buffer ROS, supply compatible solutes, alter antioxidant capacity, and influence water transport. Water-stressed wheat exudates provide microbial osmoprotectants and induce uptake pathways required for rhizosphere competence (Bhattacharyya et al., 2025), while *Trichoderma* activates host antioxidant machinery during saline stress (Brotman et al., 2013). Mycorrhizal regulation of root and leaf aquaporins provides a direct bridge from colonization to hydraulic recovery and CO₂ assimilation (Paluch-Lubawa and Polcyn, 2024; Zou et al., 2024). These mechanisms should be tested with root and leaf water potential, osmotic potential, hydraulic conductance, stomatal behavior, ROS localization, antioxidant state, photosynthesis, and growth. Grouping them by physiological function avoids treating “drought microbes” and “salt microbes” as inherently distinct categories. Redox and osmotic feedback should also be localized. Bulk antioxidant measurements cannot distinguish damaging ROS accumulation from signaling at the root surface or symbiotic interface. Compatible solutes may protect microorganisms, plants, or both, and microbial catabolism may deplete compounds intended for plant osmotic adjustment. Hydraulic benefit can arise from fungal water transport, altered aquaporin regulation, root-system architecture, or reduced shoot demand. These alternatives make single growth measurements difficult to interpret. Combining redox imaging, microbial stress mutants, solute flux, aquaporin or hydraulic assays, and organ-specific gas exchange can identify the route by which colonization changes plant performance. The strongest evidence links a microbial function to a physiological variable and then to stress tolerance, rather than inferring protection from survival alone.

### 6.4 Root-shoot systemic feedback

Root microbiota are not endpoints of shoot-derived carbon allocation. A gnotobiotic Arabidopsis system revealed a microbiota-root-shoot circuit that shifts whole-plant growth and defense under suboptimal light (Hou et al., 2021). Mycorrhiza-associated coordination of root and leaf aquaporins further shows that microbial feedback can couple root water uptake with stomatal behavior and photosynthetic recovery (Paluch-Lubawa and Polcyn, 2024). Microbial volatiles, nutrient signals, and hormone-active metabolites may also modify shoot defense and source-sink relations. Demonstrating systemic feedback requires simultaneous measurements of root colonization, shoot photosynthesis, transpiration, hormone or defense responses, and subsequent changes in root allocation or exudation. Systemic feedback may also reshape the next round of microbiome assembly. A microbial signal that improves photosynthesis changes the carbon available for roots; altered shoot defense changes hormone transport and root immune state; and improved nutrient acquisition changes starvation-induced exudation. The microbiome can therefore affect its own niche through plant physiology. Split-root and grafting experiments can determine whether a local colonizer changes outputs from an uncolonized root sector. Combining these designs with shoot gas exchange, vascular hormone measurements, and root-zone metabolomics would reveal whether systemic feedback is coordinated through carbon, hydraulic, nutrient, or immune signals. This recursive mechanism is central to the feedback framework and distinguishes it from a linear model in which plants recruit microbes and simply receive a fixed benefit. A microbial benefit is one return path in the proposed framework, but it is not by itself a closed feedback loop. A complete loop requires a microbial perturbation to change a defined plant physiological variable or root output and that altered plant state to modify subsequent microbiome assembly. Few studies resolve all steps in one time-resolved design. We therefore distinguish four evidence classes: an iterative closed loop, a bidirectional circuit demonstrated within one study, reciprocal processes supported by compatible studies, and a one-way microbial effect on the plant. For example, the microbiota–root–shoot circuit under suboptimal light links shoot-perceived stress to root bacterial community changes and reciprocal microbiota-dependent growth–defense regulation, but does not track a second round of assembly. Systemically induced root exudation shows that local microbial colonization can reprogram distal root outputs, while the resulting second-round community response remains incompletely measured. Coumarin and phosphate-starvation systems provide reciprocal modules across studies, but complete loop closure should be treated as a hypothesis until the return effect on assembly is directly tested.

### 6.5 Colonization does not equal physiological benefit

Microbial enrichment, colonization, persistence, functional activity, and plant benefit are distinct endpoints. Drought may enrich a lineage because it tolerates low water potential, yet only selected strains may improve plant performance. A SynCom may colonize a sterile substrate but fail in resident soil, and a persistent organism may be metabolically inactive under the target stress. Claims of a beneficial microbiome should therefore demonstrate a plant phenotype and identify the microbial function required for it. Necessity, sufficiency, persistence, and net physiological benefit should be measured separately and then linked through rescue or reconstruction. These distinctions should determine terminology. “Enriched” describes relative abundance, “colonized” requires verified root association, “persistent” requires temporal stability, and “functionally active” requires an expressed or measured process. “Beneficial” should be reserved for a demonstrated improvement in plant phenotype under the specified context. Even then, benefit may be conditional on dose, community background, and stress severity. A strain that improves growth under severe drought may impose a carbon or immune cost under well-watered conditions. Drought-protective effects can also be strain specific within enriched lineages, as shown for wheat-associated *Streptomyces* isolates (Si et al., 2021). Reporting all five endpoints makes negative and context-dependent results informative and aligns microbial claims with the causal evidence stages.

## 7 From causal mechanisms to physiological microbiome manipulation

Mechanistic intervention can target the physiological outputs that construct a microbial niche or use defined communities to test and deliver functions. Both routes must account for resource cost, pleiotropy, resident-community competition, and environmental variation. Manipulation is most informative when it connects a controllable plant variable to microbial decoding and a measured plant physiological outcome. Each proposed intervention should be specified as a falsifiable plant–microbe design. First, define the manipulable plant trait, including its tissue, timing and expected physiological cost. Second, define the microbial decoder trait—such as a receptor, chemotaxis module, transporter, catabolic pathway or stress-tolerance function—and perturb it genetically or chemically. Third, match the claimed benefit to a physiological endpoint rather than to growth alone. Fourth, prespecify failure criteria, including lack of establishment, loss of function after establishment, absence of plant benefit and benefit that is outweighed by carbon or immune cost. Fifth, test ecological controls that include a resident community, priority effects, soil chemistry, host genotype and realistic stress fluctuations. Finally, quantify off-target risks, including pathogen attraction, pleiotropic changes in root development or immunity, resource competition and genotype-by-environment dependence. These criteria convert the framework from a descriptive sequence into an experimentally testable design strategy.

### 7.1 Manipulating plant physiological outputs

Plant-oriented targets include signal biosynthesis, transporters, root-zone secretion, root architecture, carbon allocation, and immune calibration. Coumarin pathway genetics and sugar transporters show that host metabolism and spatial export can reproducibly alter colonization (Stringlis et al., 2018; Loo et al., 2024). Exogenous purines or nucleotides offer chemical proof of principle under salinity and heat (Zheng et al., 2024; Liu et al., 2025), but dose, timing, localization, persistence, carbon cost, and off-target use determine the outcome. Constitutive exudation may attract exploiters or disturb nutrient homeostasis. Breeding or editing should therefore target inducible and spatially controlled physiological traits while monitoring growth, pathogen susceptibility, and performance across environments (Escudero-Martinez and Bulgarelli, 2023).

### 7.2 Synthetic communities as tools for causal physiology

Synthetic communities are most valuable as causal instruments. Plant knockouts can test signal production or gating; microbial receptor, transporter, or catabolic mutants can test decoding; strain dropout can reveal keystones or distributed redundancy; and complementation or metabolite rescue can reconstruct the plant-microbe-phenotype chain. Binary association assays and cross-kingdom SynComs already show that plant outcomes can depend on interactions rather than on one isolate (Herrera Paredes et al., 2018; Durán et al., 2018). A strong design measures colonization, persistence, functional activity, and host physiology separately so that successful establishment is not mistaken for benefit. Synthetic ecosystems that include plants, bacteria, fungi, soil structure, and fluctuating stress can then test whether the reconstructed mechanism survives ecological complexity (Chesneau et al., 2025; Bai et al., 2015; Niu et al., 2017; Finkel et al., 2020; Carlström et al., 2019; Vorholt et al., 2017).

### 7.3 Ecological validation

Causal chains resolved in sterile substrate must be tested against resident communities, host genotype, soil chemistry, climate, and priority effects. Host preference and invasiveness vary among commensal bacteria, making establishment history a central determinant of outcome (Wippel et al., 2021). Nonsterile soil, multiple genotypes, sites, seasons, and field perturbations are therefore evidence at Stage 5 rather than optional translation. A desert-derived SynCom that improved tomato salt resilience in the presence of a resident soil microbiome illustrates this stronger validation step (Schmitz et al., 2022). Crop rotation and native microbiome inoculation can restore disease suppression, illustrating that community history and local adaptation matter (Zhou et al., 2023). Validation should test whether the same physiological function converges even when the responding taxa differ (Toju et al., 2018; Busby et al., 2017; Hassani et al., 2018).

### 7.4 Predictive modeling

Predictive models are useful only when grounded in manipulable plant physiological variables. Genome-resolved and metabolic models can nominate transport, catabolic, competition, and cross-feeding mechanisms, but prediction should be tested with physiological perturbations and plant phenotypes (Xu et al., 2021; Mataigne et al., 2022). AI does not replace causal labels: inconsistent metadata on host genotype, soil, development, exudation, colonization, and plant function currently limit transfer. The immediate priority is interoperable experiments that pair plant-state measurements with microbial mutants, community reconstruction, and realistic environmental validation (Ke et al., 2021).

## 8 Mechanistic gaps and experimental priorities

### 8.1 Spatial resolution of physiological encoding

Root tips, elongation zones, mature tissues, lateral-root junctions, and root hairs differ in permeability, transport, immune competence, and contact with soil pores. Sugar transporters already provide causal evidence that plant physiology can spatially organize colonization along the root axis (Loo et al., 2024), and root hair regulators connect developmental anatomy to drought-associated microbiome feedback (Wang et al., 2024). The next question is whether defined cell types and root zones release distinct signal combinations at concentrations that microorganisms experience in soil. Spatial metabolomics, isotope tracing, microsensors, microbial reporters, and root-zone-specific perturbations are needed to connect local plant physiology with microbial decoding and whole-plant benefit.

### 8.2 Temporal dynamics and causal ordering

Stress duration, developmental stage, circadian phase, and recovery alter both plant outputs and microbial activity. Exudate profiles show diurnal signatures and are sensitive to collection duration (McLaughlin et al., 2023), while drought can change microbiome succession over days and leave persistent legacies after rewatering. Time-resolved experiments should distinguish rapid physiological encoding from slower growth, tissue damage, and community turnover. Sampling must be frequent enough to test whether a signal precedes microbial response and whether microbial feedback precedes improved physiology. Temporal rescue experiments can determine whether the same intervention is effective during stress onset, acclimation, or recovery.

### 8.3 Causal reconstruction across plant and microbial partners

Few systems reconstruct the complete signal chain in both partners. The decisive experiment pairs a plant perturbation in biosynthesis, transport, development, or immune state with a microbial perturbation in sensing, movement, uptake, or metabolism, followed by rescue of colonization and plant phenotype. Exudate transfer should be separated from live-root effects, and microbial enrichment should be distinguished from persistence and functional activity. Physiological readouts must match the proposed benefit: nutrient flux for nutrient acquisition, hydraulic and osmotic measurements for water-stress protection, hormone reporters for growth-defense balance, and redox imaging for antioxidant feedback. The five causal evidence stages provide a common framework for these experiments.

### 8.4 Ecological generality and transferability

Plant control is conditional rather than absolute. Soil physicochemistry defines the regional species pool and the diffusion and persistence of root outputs; dispersal, priority effects, antagonism, and resident-community metabolism determine which responders retain access to the niche. Similar plant physiological states may therefore recruit different taxa across soils while producing convergent functions such as iron acquisition, osmoprotection, or redox buffering. Generality should be tested across genotypes, soils, climates, and management histories, with explicit comparison of taxonomic and functional convergence. Predictive models can help prioritize experiments, but they are useful only when grounded in manipulable physiological variables and causal labels. Common metadata should include plant carbon, water, nutrient, redox and hormone state, root-zone output measurements, microbial sensor or metabolic genes, colonization, persistence, and net plant benefit (Fitzpatrick et al., 2018; Thiergart et al., 2019). Field generality will rarely mean taxonomic identity. A plant output can be decoded by different resident organisms that share transport, metabolic, or stress-response traits. Conversely, the same taxon can behave differently when community partners, soil chemistry, or host genotype change. Multi-environment tests should therefore include both strain-resolved and functional measurements and should quantify establishment probability, redundancy, and net plant effect. Locally adapted communities may outperform universal inoculants even when both carry the same nominal function. The most transferable prediction may be that a defined plant physiological state creates a functional niche, not that it recruits one named organism. This framing preserves the conditional boundary of plant control while allowing mechanistic knowledge to guide physiological manipulation.

## 9 Conclusion

Environmental stress shapes root microbiomes through both direct environmental filtering and plant-mediated niche construction. Plant physiological state can bias microbial assembly through root outputs and immune-metabolic gating, but the strength of evidence differs among association, colonization, persistence, functional activity, plant benefit and ecological generality. Chemically defined symbioses and coumarin-mediated selection provide comparatively strong causal modules, whereas many stress-associated primary metabolites remain candidate cues, resources or osmoprotectants. Microbial effects on plant nutrition, hormones, redox balance, hydraulics and defense establish important return paths, but complete iterative feedback loops remain uncommon. A causal-evidence framework linking physiological encoding, microbial decoding and feedback therefore provides testable criteria for identifying where causal chains are complete, where environmental filtering offers an alternative explanation and where the next decisive experiment is needed.
